# A Diagnostic Panel of DNA Methylation Biomarkers for Lung Adenocarcinoma

**DOI:** 10.3389/fonc.2019.01281

**Published:** 2019-12-03

**Authors:** Nan Shen, Jun Du, Hui Zhou, Nan Chen, Yi Pan, Jörg D. Hoheisel, Zonghui Jiang, Ling Xiao, Yue Tao, Xi Mo

**Affiliations:** ^1^Department of Infectious Diseases, Shanghai Children's Medical Center, Shanghai Jiao Tong University School of Medicine, Shanghai, China; ^2^Pediatric Translational Medicine Institute, Shanghai Children's Medical Center, Shanghai Jiao Tong University School of Medicine, Shanghai, China; ^3^Diagnostic Imaging Center, Shanghai Children's Medical Center, Shanghai Jiao Tong University School of Medicine, Shanghai, China; ^4^Lymphoma & Hematology Department, Tumor Hospital of Xiangya School of Medicine of Central South University, Changsha, China; ^5^Xinhua Hospital Affiliated to Shanghai Jiao Tong University School of Medicine, Chongming Branch, Shanghai, China; ^6^Division of Functional Genome Analysis, German Cancer Research Center (DKFZ), Heidelberg, Germany; ^7^Faculty of Medicine Heidelberg, Heidelberg University, Heidelberg, Germany; ^8^Department of Medical Oncology, The First People's Hospital, Chuzhou, China; ^9^Department of Histology and Embryology of School of Basic Medical Science, Central South University, Changsha, China

**Keywords:** lung adenocarcinoma, DNA methylation, random forest, *HOXA9*, *KRTAP8-1*, *CCND1*, *TULP2*, biomarker

## Abstract

Lung adenocarcinoma (LUAD) is one of the most common cancers and lethal diseases in the world. Recognition of the undetermined lung nodules at an early stage is useful for a favorable prognosis. However, there is no good method to identify the undetermined lung nodules and predict their clinical outcome. DNA methylation alteration is frequently observed in LUAD and may play important roles in carcinogenesis, diagnosis, and prediction. This study took advantage of publicly available methylation profiling resources and a machine learning method to investigate methylation differences between LUAD and adjacent non-malignant tissue. The prediction panel was first constructed using 338 tissue samples from LUAD patients including 149 non-malignant ones. This model was then validated with data from The Cancer Genome Atlas database and clinic samples. As a result, the methylation status of four CpG loci in homeobox A9 (*HOXA9*), keratin-associated protein 8-1 (*KRTAP8-1*), cyclin D1 (*CCND1*), and tubby-like protein 2 (*TULP2*) were highlighted as informative markers. A random forest classification model with an accuracy of 94.57% and kappa of 88.96% was obtained. To evaluate this panel for LUAD, the methylation levels of four CpG loci in *HOXA9, KRTAP8-1, CCND1*, and *TULP2* of tumor samples and matched adjacent lung samples from 25 patients with LUAD were tested. In these LUAD patients, the methylation of *HOXA9* was significantly upregulated, whereas the methylation of *KRTAP8-1, CCND1*, and *TULP2* were downregulated obviously in tumor samples compared with adjacent tissues. Our study demonstrates that the methylation of *HOXA9, KRTAP8-1, CCND1*, and *TULP2* has great potential for the early recognition of LUAD in the undetermined lung nodules. The findings also exhibit that the application of improved mathematic algorithms can yield accurate and particularly robust and widely applicable marker panels. This approach could greatly facilitate the discovery process of biomarkers in various fields.

## Introduction

Lung cancer is the leading cause of cancer-related deaths worldwide. As the most frequent histological subtype of non-small-cell lung cancer (NSCLC), lung adenocarcinoma (LUAD) accounts for more than 40% of the incidence of lung cancer. It is usually diagnosed at an advanced stage. When diagnosed at an early stage, however, the survival rate is dramatically prolonged. The 1-year survival rate of stage I NSCLC is 81–85%, while it is only 15–19% when diagnosed at stage IV (1). Effective early recognition methods and relevant biomarkers are urgently needed to reduce the mortality caused by LUAD. Traditional screen methods for NSCLC include sputum cytology, chest radiography, and computed tomography (CT). CT, which is simple and with high sensitivity, has been shown to be capable of detecting early stage lung cancers and is beneficial for the treatment and patient outcome. Although high false-positive rate has been reported to be a major problem in LUAD by CT, several studies have shown that CT-positive findings that are largely based on nodule size and/or volume can reduce false-positive rates and the risk of overdiagnosis (2). In addition, due to the demand for further optimization of CT screening in LUAD, there is still a need for other available biomarkers to support the risk assessment (3). For example, a number of changes in gene expression (4, 5), somatic mutations (6), copy number variations (7), differences in methylation (8) or the abundance of plasma proteins (9), and sequence-variations in circulating free DNAs (10, 11) have been extensively studied. In recent years, aberrant changes in DNA methylation have been observed in various cancers and are considered to be a cause of tumorigenesis. Global hypomethylation occurs frequently in repeated DNA sequences and plays an important role in chromosomal instability. The promoter regions of tumor suppressor genes are often hypermethylated, leading to inactivation of corresponding genes in tumors. It has been reported that *p16* (12), *APC* (13), *BCL2* (14), *BRCA1*, and *BRCA2* (15) are hypermethylated in NSCLC, but single methylation markers and the bias introduced by experiments often caused these methods to suffer problems of insufficient precision and specificity. To improve the detection performance of methylation biomarkers, many panels of multiple loci were studied (16, 17). DNA methylation is a relatively stable biochemical modification carried out by DNA methyltransferases and can be detected in not only DNA molecules from tissue but also the free DNAs in serum and plasma (18), making it a promising biomarker for the early recognition of the undetermined lung nodules.

With the advent of big data areas, publicly available databases such as The Cancer Genome Atlas (TCGA), The Encyclopedia of DNA Elements, and Gene Expression Omnibus (GEO) contain many methylation profiles for a variety of tumors and normal samples. The development of machine learning technology and its application in the biological fields makes it possible to select the most inspective detection markers from a massive number of potential loci and establish prediction models with better performance.

In this study, we utilized methylation array data from publicly available databases and tried to explain lung carcinogenesis from an epigenetic perspective. Because early recognition of LUAD can benefit patients, we took advantage of machine learning algorithms to build a concise but robust prediction model using methylation markers for making predictions on patients with lung cancer. As a result, the methylation status of four CpG loci in homeobox A9 (*HOXA9*), keratin-associated protein 8-1 (*KRTAP8-1*), cyclin D1 (*CCND1*), and tubby-like protein 2 (*TULP2*) were highlighted as potential biomarkers. Then, we identified four LUAD-specific methylation biomarkers by comparing LUAD tumor samples and matched adjacent samples. The results further confirmed that the hypermethylation of *HOXA9* and the hypomethylation of *KRTAP8-1, CCND1*, and *TULP2* were observed in LUAD tumor samples. This study could also pave ways for a more extensive application of non-invasive cancer detection.

## Materials and Methods

### Patients and Clinical Samples

A total of 25 patients with LUAD were recruited. This study was carried out in accordance with the 1964 Declaration of Helsinki and its later amendments with written informed consent from all subjects. The study was approved by the Institutional Review Board and the Ethics Committee of the Affiliated Tumor Hospital of Xiangya Medical School of Central South University and Xinhua Hospital Affiliated to Shanghai Jiao Tong University School of Medicine, Chongming Branch. Tumor samples and matched adjacent lung samples from 25 patients with LUAD were obtained. None of patients had undergone ablation, chemotherapy, or radiotherapy before resection. The clinical and pathological characteristics of 25 patients with LUAD are summarized in Supplementary Table 1.

### Datasets

In the discovery phase, data about 338 LUADs and non-malignant samples were collected from GEO datasets that are based on analyses with the Illumina Infinium HumanMethylation27 BeadChip for DNA methylation measurements. Detailed dataset descriptions are shown in Table 1. TCGA LUAD validation cohorts profiled by both the Illumina Infinium HumanMethylation27 and HumanMethylation450 platforms were downloaded from Xena Functional Genomics Explorer. These datasets are heavily imbalanced. For analyses with the Illumina Infinium HumanMethylation27 system, there are only 24 normal samples out of a total of 151 samples, and for the HumanMethylation450 platform, there are only 34 normal samples within the total of 492 analyzed samples.

**Table 1:** Summary of the gene expression omnibus (GEO) datasets.

**Sample ID**	**Tumor**	**Normal**
GSE32861	59	59
GSE32866	28	27
GSE62948	28	28
GSE63384	35	35
GSE83845	39	0
**Total**	**189**	**149**

### Identification of Differentially Methylated Probes

The 338 LUADs and non-malignant samples in the GEO database profiled by Illumina Infinium HumanMethylation27 BeadChip were used for differential methylation analysis. The datasets from the five experiments were combined using R package illuminaio by common probe IDs. Array data produced by different studies are confounded by non-biological variables such as different technicians or the environments. These biases are termed as “batch effects” and cannot be eliminated unless all the samples are performed in a single batch. Here, we adjusted the batch effects by applying gene-wise one-way ANOVA adjustment for expression values using the pamr.batchadjust function from the pamr package. Differential methylation analysis was performed using linear model provided in the limma R package (19). Probes that were differentially methylated between LUADs and non-malignant tissues were selected with thresholds of |logFC| > 1 and adjusted *p* < 0.05. Gene oncology and Reactome pathway enrichment analysis were performed using DAVID version 6.8 (20).

### Building of a Prediction Model

Random forest was chosen as the prediction model because of its superior performance for classification with binary features. To make a robust, easy-to-generalize model to predict the sample status, each of the differentially methylated probes was first binary assigned. First K-means clustering with two cores was applied to each probe, and the mean values were calculated for both clusters. Samples in the high/low mean cluster were signed as 1 or 0, respectively. In the training phase, the importance value of each probe to the classification model was evaluated by recursive feature elimination. According to descending importance value, the selected CpGs were added one by one to the random forest model if its Pearson correlation value with any already existing probe in the model was <0.7. Each time a new feature was added to the model, the performance of the model was re-evaluated using 10-fold cross-validation. The final model was chosen when best accuracy and kappa were achieved.

### Model Validation

In the validation phase, four candidate CpGs were validated using two TCGA cohorts. LUAD methylation status was profiled by two platforms, namely, the HumanMethylation27 and HumanMethylation450 systems. Since both platforms contain all four selected probes, both the above two datasets were used for model validation. LUAD methylation datasets profiled by Illumina Infinium HumanMethylation27 platform had a total of 151 samples, with 127 from LUADs and 24 from non-malignant samples, and the data from the HumanMethylation450 platform consisted of 492 samples, which contained 458 samples from tumors and only 34 from normal tissues. Owing to the batch effect mentioned above, K-means clustering was recalculated for the four selected CpG probes in the TCGA HumanMethylation27 and HumanMethylation450 datasets, respectively, to reassign the binary methylation levels for each probe in all samples. For both datasets, 80% samples were randomly extracted as training set, while the remaining 20% samples were set aside for performance testing separately. Because of the heavy imbalance of the datasets, in the training phase, random oversampling examples (ROSEs) were applied to make non-malignant samples accounting for 50% of the training set to improve the model performance.

### DNA Extraction and Methylation Analysis by Pyrosequencing of Patients' Samples

QIAamp DNA Mini Kit was used for DNA extraction according to the manufacturer's instructions. A total of 500 ng of DNA was converted using the EZ DNA Methylation-Gold (Zymo Research Corporation, Irvine, USA) bisulfite conversion kit following the manufacturer's recommendations. Specific sets of primers for polymerase chain reaction (PCR) amplification and sequencing were designed and synthetized by Sangon Biotech (Shanghai, China). Primer sequences were designed, when possible, to hybridize with CpG-free sites to ensure methylation-independent amplification. PCR was performed under standard conditions with biotinylated primers, and the PyroMark Vacuum Prep Tool (Biotage AB, Uppsala, Sweden) was used to prepare single-stranded PCR products, according to the manufacturer's instructions. Forty microliter of DNA PCR products was added to a mixture consisting of 0.5 μM sequencing primers, Sepharose beads (GE Healthcare) and binding buffer, and mixed for 5 min at room temperature. A vacuum prep workstation and PyroMark Gold Q96 system (Qiagen) were used to perform pyrosequencing reactions according to the manufacturer's instructions. CpG site quantification was analyzed with the PyroMark CpG Software 1.0.11.

### Statistical Analysis

All statistical analysis was performed with R version 3.0.2, and the graphs were generated using GraphPad PRISM 7.0 (GraphPad Software, Inc., San Diego, CA, USA). The differential methylation levels of tumors and non-malignant samples in patients with LUADs were compared using *t* test. A *p* < 0.05 was considered statistically significant.

## Results

### Datasets and Differential Methylation Analysis

Four GEO datasets produced on Illumina Infinium HumanMethylation27 BeadChips for LUAD methylation profiling with a total of 338 samples (189 tumor samples and 149 matched adjacent lung samples) were included in our study. The analysis identified 62 probes that were differentially methylated between LUADs and adjacent non-malignant lung tissues. Among them, only 3 were hypomethylated in tumor samples, while all other 59 probes showed a hypermethylated status (Table 2).

**Table 2:** Differentially methylated probes detected in the Gene Expression Omnibus (GEO) datasets.

**ID**	**Symbol**	**logFC**	**Adjusted p-value**	**ID**	**Symbol**	**logFC**	**Adjusted p-value**
cg26521404	*HOXA9*	1.41	7.07E−24	cg04490714	*SLC6A2*	1.03	3.67E−17
cg01354473	*HOXA9*	1.12	6.87E−23	cg01009664	*TRH*	1.08	4.21E−17
cg25720804	*TLX3*	1.42	8.39E−23	cg25875213	*ZNF781*	1.37	1.05E−16
cg15540820	*EOMES*	1.04	2.58E−20	cg01295203	*PRDM14*	1.04	1.28E−16
cg22660578	*LHX1*	1.10	3.78E−20	cg14458834	*HOXB4*	1.32	1.80E−16
cg01381846	*HOXA9*	1.01	4.57E−20	cg07307078	*TUBB6*	1.07	3.09E−16
cg12374721	*PRAC1*	1.17	4.57E−20	cg12680609	*ZFP41*	1.02	5.88E−16
cg08089301	*HOXB4*	1.55	6.89E−20	cg06151165	*VSX1*	1.07	6.28E−16
cg23290344	*NEFM*	1.28	7.04E−20	cg05436658	*PRKCB*	1.29	1.00E−15
cg07533148	*TRIM58*	1.54	7.04E−20	cg17619823	*ADRB3*	1.09	1.85E−15
cg12880658	*CDO1*	1.29	7.96E−20	cg26963271	*PDE4B*	1.04	2.25E−15
cg19456540	*SIX6*	1.42	2.06E−19	cg21529533	*HLA*–*G*	1.06	3.08E−15
cg13323752	*SLC2A14*	1.34	2.31E−19	cg10303487	*DPYS*	1.13	3.28E−15
cg04534765	*GALR1*	1.23	2.50E−19	cg25691167	*FERD3L*	1.14	3.31E−15
cg08118311	*SALL3*	1.19	2.67E−19	cg26721264	*GALR1*	1.05	3.40E−15
cg02164046	*SST*	1.02	2.92E−19	cg21546671	*HOXB4*	1.16	3.76E−15
cg14859460	*GRM6*	1.09	3.29E−19	cg25574024	*NA*	1.02	4.55E−15
cg02008154	*TBX20*	1.00	3.96E−19	cg15520279	*HOXD8*	1.04	1.38E−14
cg18952647	*BNC1*	1.30	4.06E−19	cg00949442	*ABCA3*	1.02	2.37E−14
cg07778029	*HOXA9*	1.05	4.13E−19	cg09516965	*PTGDR*	1.26	2.91E−14
cg20959866	*AJAP1*	1.05	1.32E−18	cg16731240	*ZNF577*	1.04	3.74E−14
cg22471346	*GAS7*	1.36	2.02E−18	cg10883303	*HOXA13*	1.03	4.78E−14
cg14991487	*HOXD9*	1.21	4.14E−18	cg13912117	*ADCY8*	1.05	5.27E−14
cg22881914	*NID2*	1.42	4.55E−18	cg00848728	*DAB1*	1.09	5.27E−14
cg24423088	*KRTAP8-1*	−1.07	4.72E−18	cg21790626	*ZNF154*	1.13	1.20E−13
cg23432345	*HOXA7*	1.20	4.72E−18	cg01805540	*CACNB2*	1.01	3.98E−12
cg06760035	*HOXB4*	1.40	5.67E−18	cg09229912	*CUX2*	1.06	4.84E−12
cg18722841	*PHOX2A*	1.04	5.68E−18	cg18349835	*VIPR2*	1.10	6.70E−12
cg15191648	*SALL3*	1.04	6.75E−18	cg00062776	*TULP2*	−1.00	2.08E−11
cg04048259	*EDN3*	1.10	1.32E−17	cg19332710	*RIMS4*	1.00	1.67E−10
cg17525406	*AJAP1*	1.22	1.90E−17	cg02723533	*CCND1*	−1.04	2.70E−10

Gene oncology (GO) and Reactome pathway enrichment analysis were carried out using DAVID24 (adjusted *p* < 0.05, Table 3). GO enrichment results identified items related to transcription factor activity, indicating that methylation can change gene expression in tumor samples by modulating the activities of transcription factors. Both GO and pathway analysis showed the enrichment in G-protein-coupled receptor signaling pathway, which is often dysregulated in tumor cells to facilitate their proliferation, invasion, and immune system escape.

**Table 3:** Gene Ontology (GO) and Kyoto Encyclopedia of Genes and Genomes (KEGG) pathway enrichment results of differentially methylated genes.

**Term**	**Count**	**Benjamini**
**BIOLOGICAL PROCESS**		
Negative regulation of transcription from RNA polymerase II promoter	10	2.90E−02
Transcription, DNA-templated	17	3.60E−02
Adenylate cyclase-activating G-protein coupled receptor signaling pathway	4	4.40E−02
**CELL COMPONENT**		
Nucleus	28	2.80E−02
**MOLECULAR FUNCTION**		
Sequence-specific DNA binding	11	2.10E−04
RNA polymerase II regulatory region sequence-specific DNA binding	8	1.20E−04
Transcription factor activity, sequence-specific DNA binding	12	2.80E−03
**REACTOME PATHWAYS**		
G alpha (s) signaling events	5	3.00E−02

### Feature Selection and Construction of the Prediction Panel

**Figure 1:**
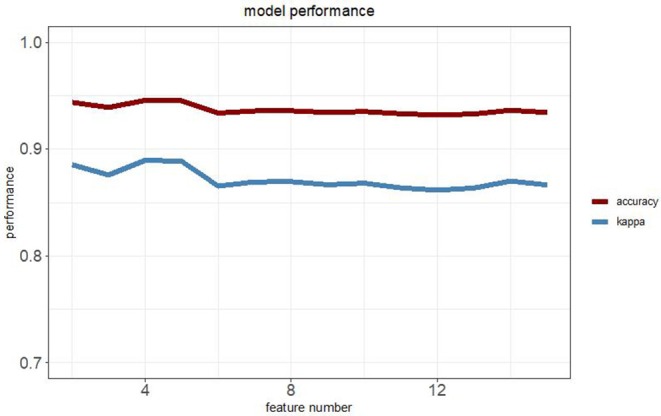
Relationship between the model performance and the feature numbers based on both accuracy and kappa value.

**Figure 2:**
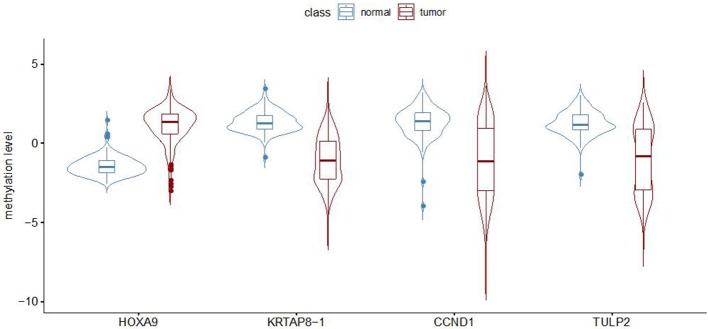
Methylation level (beta value) for the four selected probes in lung adenocarcinomas and non-malignant samples from GEO dataset measured by Illumina Infinium HumanMethylation27 BeadChip.

To establish a robust prediction model to identify LUADs, we binarily assigned each differentially methylated probe using K-means clustering algorithms. Recursive feature elimination was applied to evaluate the discriminative power of methylation loci to ensure that only the most informative features could be included in the final prediction model. The correlation assessment between loci was also used when adding loci one by one during model construction to ensure that the loci with relatively small effect were set by removing possible redundant information. Each time a new probe was added to the model, the prediction performance was updated with 10-fold cross-validation. The eventual model was chosen based on both accuracy and kappa values (Figure 1). Finally, a random forest classification model with an accuracy of 94.57% and a kappa of 88.96% was obtained. The methylation status of four CpG loci of the genes of homeobox A9 (*HOXA9*), *KRTAP8-1, CCND1*, and *TULP2* were highlighted as predictors in the final model. Methylation levels of the selected probes between adenocarcinomas and normal tissues are shown in Figure 2.

### Validation of the Prediction Model

**Figure 3:**
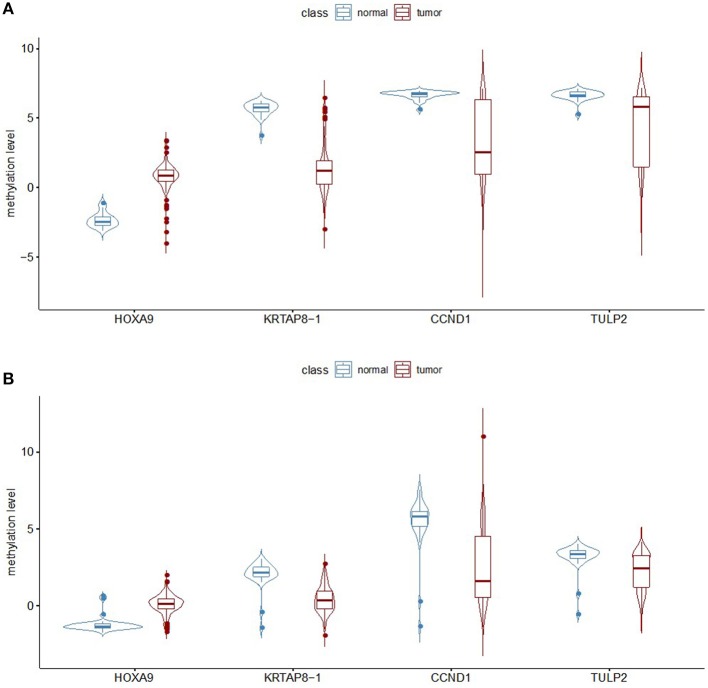
Analysis of the methylation level of the four selected probes. **(A)** The beta value for the four selected probes was calculated from the TCGA dataset representing lung adenocarcinomas and non-malignant samples, with the measurements being done on the Illumina Infinium HumanMethylation27 BeadChip platform. **(B)** Analysis of the TCGA data generated on the Illumina Infinium HumanMethylation450 BeadChip platform.

This model was then validated in two independent TCGA LUAD methylation datasets profiled on both the Illumina Infinium HumanMethylation27 platform and HumanMethylation450 platform. The methylation patterns of the four selected probes are the same between the GEO and TCGA datasets (Figure 3). There are 151 samples in the TCGA LUAD HumanMethylation27 dataset, with 127 from LUADs and 24 from adjacent non-malignant lung tissues. Imbalanced datasets in which the sample number in one class outnumbers the other class by a large proportion often yield a prediction model of reduced overall accuracy and particularly bias performance with respect to the minority group. Here, we used ROSE to generate an artificially balanced training dataset based on sampling methods and smoothed bootstrap approach. By randomly sampling 80% samples as training dataset, the random forest predication model with the selected four CpG loci can achieve an accuracy of 96.55% and kappa of 86.88%. As all the four selected loci are also covered by the HumanMethylation450 platform, we checked the prediction power of the above probes in the TCGA LUAD HumanMethylation450 dataset. There are 492 samples in this dataset, among which 458 are from LUADs. We used ROSE again to generate a balanced training set with 80% of all samples and tested the model performance with the remaining 20% samples. This model achieved an accuracy of 94.85% and kappa of 63.99%.

**Figure 4:**
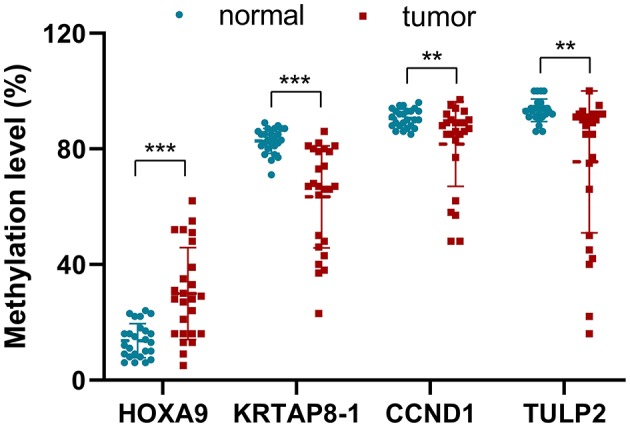
The methylation level (%) for the four selected probes of the tumors and non-malignant samples validated in 25 patients with LUAD using pyrosequencing (**p < 0.001, ***p < 0.0001).

To evaluate the relevance of this prediction model further, DNA methylation analysis of these four selected probes was performed in 25 paired samples, including LUAD and matched non-malignant tissues, from patients with LUAD collected by two hospitals using pyrosequencing (Figure 4). Although the sample size in this study was limited, the analysis revealed congruence with the GEO and TCGA data. The methylation level of *HOXA9* in tumors was upregulated compared to non-malignant samples (29.96 ± 15.89% vs. 13.64 ± 5.89%, *p* = 0.00007). On the other hand, the methylation level of *KRTAP8-1* (63.40 ± 17.62% vs. 82.76 ± 4.32%, *p* = 0.00002), *TULP2* (81.68 ± 14.63% vs. 90.56 ± 3.19%, *p* = 0.00837), and *CCND1* (75.52 ± 24.55% vs. 93.36% ± 3.89%, *p* = 0.00012) were downregulated in tumors than that in normal samples.

## Discussion

Epigenetic markers such as DNA methylation and histone modifications may be used as candidate biomarkers for classifying individuals with respect to their disease risk. Along with the wide application of high-throughput epigenomic measurement technologies and the resulting publicly available databases, more and more big data-based biomarkers have been proposed. Recently, the incidence of small nodules in the lungs stays high worldwide. How to distinguish between benign and malignant nodules is a clinical problem that needs to be solved urgently. To develop a reliable recognition panel utilizing DNA methylation signals, we selected training samples of tumors and non-malignant tissues from the GEO dataset to build a predication model for LUAD and tested our prediction model in two validation sets derived from the TCGA database. In this study, we systematically analyzed the DNA methylation data of LUAD and found that the hypermethylation of *HOXA9* and the hypomethylation of *KRTAP8-1, CCND1*, and *TULP2* were observed in LUAD tumor samples. This study identified that the methylation levels of *HOXA9, KRTAP8-1, CCND1*, and *TULP2* may be helpful as LUAD-specific diagnostic panel in undetermined lung nodules.

Researchers have worked in the field of identifying methylation panels, which combine multiple genes for cancer detection as well as prognosis prediction. Ooki et al. proposed a methylation panel of six genes (*CDO1, HOXA9, AJAP1, PTGDR, UNCX*, and *MARCH11*) with a prediction accuracy of 92.2% in the training cohort and 93.0% in an independent testing cohort of stage IA primary NSCLC (21). Another study showed that hypermethylation of five genes (*HIST1H4F, PCDHGB6, NPBWR1, ALX1*, and *HOXA9*) was significantly associated with shorter relapse-free survival in stage I NSCLC (22). However, these studies mainly followed the path of directly combining altered methylation loci with known biological functions related to cancer. Such approach ignores the potential correlations between selected features, which will in turn result in marker redundancy. In addition, directly using methylation values as model input makes the model both vulnerable and difficult to generalize. As more and more extensive applications of machine learning techniques enter the fields of biomarker discovery and prediction model building, we took advantage of unsupervised clustering method for a binary feature transforming and recursive feature elimination to select the most concise but simultaneously informative methylation marker set. The random forest machine learning model, which shows superior performance in the tasks of binary classification, was applied in the present study for model construction. Random forest is a useful algorithm, which can select featured genes differentially expressed among different samples. The advantage of random forest is that it can avoid overfitting effectively although the mechanism is not currently clear (23). In addition, we used a value of bagged trees sufficiently large to further settle down the error rate in the present study. In this study, we have identified a candidate prediction panel of four CpG loci located in *HOXA9, KRTAP8-1, CCND1*, and *TULP2*, which achieves an accuracy of 94.57%. The prediction panel could be validated in three independent testing cohorts, including two large datasets from TCGA and one experimental dataset. Remarkably, the results were similar among different datasets and were not affected by the platform used in the analysis. The results demonstrated that the application of random forest machine learning model could greatly improve the ability to discovery cancer biomarkers.

Of the four selected methylation markers, *HOXA9* and *CCND1* have previously been reported to participate in the occurrence and development of tumors. Nelson et al. identified a 10.3-fold higher level of methylation of *HOXA9* in lung tumor than that in the adjacent normal tissues in 146 sample pairs (24). Other studies including 40 pairs of primary lung cancer and normal tissues as well as 185 induced sputum specimens also found that methylation of *HOXA9* in lung cancer tissues was significantly higher compared with normal tissues. Further investigation discovered that the *HOXA9* gene in lung cancer patients were significantly more hypermethylated compared with patients with benign lung diseases and the healthy group (25). In addition to *HOXA9*, the methylation of *HOXB4, HOXD8*, and *HOXD9* were also different between tumor and adjacent normal tissues in lung cancer (Table 2). *HOX* genes play crucial roles in a wide range of processes, including in lung development and are expressed in the normal adult lungs. Hence, abnormal expression or methylation levels of *HOX* gene might cause lung cancer (26). In addition to *HOXA9, CCND1* has long been described as a prognosis maker for NSCLC. It plays key roles in G1/S phase transition and can promote tumor cell proliferation. Studies on 69 resected NSCLC samples between stages I and IIIA showed that overexpression of *CCND1* is significantly positively correlated with lymph nodes metastasis, advanced pathological stages, and poor survival (27). As opposed to *HOXA9* and *CCND1*, there is no report on changes in the methylation status in cancers of *KRTAP8-1* and *TULP2*. The underlying mechanisms, by which methylation of these two genes changes the biology in tumors, need further investigation.

Although the prediction panel demonstrates high accuracy in both TCGA datasets, the present study still has some limitations. First, all data used were from methylation arrays. Nowadays, more and more high-throughput methylation detection methods such as reduced representation bisulfite sequencing (28) and methyl-sensitive cut counting technique (29) have been applied to both scientific and clinical examinations. An adjustment of our model for fitting a wider range of applications may be required. Second, we only studied our model in LUAD tumors and non-malignant tissues. Thus, further analysis that aims to validate this prediction panel in the body fluids such as blood samples from lung cancer patients for non-invasive diagnostics will be required. Third, markers or marker panels for a more accurate prediction of NSCLC subtypes are also in urgent need to provide guidance for more personal and precise treatment and prognosis. Lastly, the prediction panel in this study was only validated in patients with LUAD. However, standard validation should be performed in the clinical setting of an intended-to-use cohort (3). Therefore, the efficacy of the prediction panel will be investigated in multiple cohorts such as patients with undetermined lung nodules in the future study. In summary, this study presents a process by which robust and accurate diagnostic panels can be obtained. It was applied to methylation data from lung cancer patients and relevant control samples. The study demonstrated the effectiveness of the procedure, which could be applied to different sample cohorts and diseases other than cancer, thereby greatly facilitating the discovery process of biomarkers in various fields. For lung cancer, it was shown that the methylation levels of *HOXA9, KRTAP8-1, CCND1*, and *TULP2* have great potential for the early recognition of LUAD in the undetermined lung nodules. Their suitability for liquid biopsy still needs to be demonstrated.

## Data Availability Statement

Publicly available datasets were analyzed in this study. These data can be found here: https://www.ncbi.nlm.nih.gov/gds and https://portal.gdc.cancer.gov.

## Ethics Statement

The study was approved by the Institutional Review Board and the Ethics Committee of the Affiliated Tumor Hospital of Xiangya Medical School of Central South University and Xinhua Hospital Affiliated to Shanghai Jiao Tong University School of Medicine, Chongming Branch, and written informed consent was obtained from each patient.

## Author Contributions

XM, YT, LX, NC, and NS participated in the study design, data collection, analysis of data, and preparation of the manuscript. JD, HZ, YP, and ZJ carried out the experimental work and the data collection. JH participated in the interpretation of data and drafted the manuscript. All authors read and approved the final manuscript.

### Conflict of Interest

The authors declare that the research was conducted in the absence of any commercial or financial relationships that could be construed as a potential conflict of interest.

## References

[1] Blandin Knight S Crosbie PA Balata H Chudziak J Hussell T Dive C. Progress and prospects of early detection in lung cancer. Open Biol. (2017) 7:170070. 10.1098/rsob.170070 28878044 PMC5627048

[2] Seijo LM Peled N Ajona D Boeri M Field JK Sozzi G. Biomarkers in lung cancer screening: achievements, promises, and challenges. J Thorac Oncol. (2019) 14:343–57. 10.1016/j.jtho.2018.11.023 30529598 PMC6494979

[3] Peled N Ilouze M. Screening for lung cancer: what comes next? J Clin Oncol. (2015) 33:3847–8. 10.1200/JCO.2015.63.1713 26304887

[4] Spira A Beane JE Shah V Steiling K Liu G Schembri F. Airway epithelial gene expression in the diagnostic evaluation of smokers with suspect lung cancer. Nat Med. (2007) 13:361–6. 10.1038/nm1556 17334370

[5] Silvestri GA Vachani A Whitney D Elashoff M Porta Smith K Ferguson JS. A bronchial genomic classifier for the diagnostic evaluation of lung cancer. N Engl J Med. (2015) 373:243–51. 10.1056/NEJMoa1504601 25981554 PMC4838273

[6] Ding L Getz G Wheeler DA Mardis ER McLellan MD Cibulskis K. Somatic mutations affect key pathways in lung adenocarcinoma. Nature. (2008) 455:1069–75. 10.1038/nature07423 18948947 PMC2694412

[7] Ni X Zhuo M Su Z Duan J Gao Y Wang Z. Reproducible copy number variation patterns among single circulating tumor cells of lung cancer patients. Proc Natl Acad Sci USA. (2013) 110:21083–8. 10.1158/1538-7445.AM2014-3577 24324171 PMC3876226

[8] Palmisano WA Divine KK Saccomanno G Gilliland FD Baylin SB Herman JG. Predicting lung cancer by detecting aberrant promoter methylation in sputum. Cancer Res. (2000) 60:5954–8. 11085511

[9] Xiao T Ying W Li L Hu Z Ma Y Jiao L. An approach to studying lung cancer-related proteins in human blood. Mol Cell Proteom. (2005) 4:1480–6. 10.1074/mcp.M500055-MCP200 15970581

[10] Pathak AK Bhutani M Kumar S Mohan A Guleria R. Circulating cell-free DNA in plasma/serum of lung cancer patients as a potential screening and prognostic tool. Clin Chem. (2006) 52:1833–42. 10.1373/clinchem.2005.062893 16423903

[11] Yoon KA Park S Lee SH Kim JH Lee JS. Comparison of circulating plasma DNA levels between lung cancer patients and healthy controls. J Mol Diagn. (2009) 11:182–5. 10.2353/jmoldx.2009.080098 19324991 PMC2671334

[12] Tuo L Sha S Huayu Z Du K P16(INK4a) gene promoter methylation as a biomarker for the diagnosis of non-small cell lung cancer: an updated meta-analysis. Thorac Cancer. (2018) 9:1032–40. 10.1111/1759-7714.12783 29927090 PMC6068431

[13] Kim DS Cha SI Lee JH Lee YM Choi JE Kim MJ. Aberrant DNA methylation profiles of non-small cell lung cancers in a Korean population. Lung Cancer. (2007) 58:1–6. 10.1016/j.lungcan.2007.04.008 17532092

[14] Nagatake M Osada H Kondo M Uchida K Nishio M Shimokata K. Aberrant hypermethylation at the bcl-2 locus at 18q21 in human lung cancers. Cancer Res. (1996) 56:1886–91. 8620509

[15] Lee MN Tseng RC Hsu HS Chen JY Tzao C Ho WL. Epigenetic inactivation of the chromosomal stability control genes BRCA1, BRCA2, and XRCC5 in non-small cell lung cancer. Clin Cancer Res. (2007) 13:832–8. 10.1158/1078-0432.CCR-05-2694 17289874

[16] Dammann R Strunnikova M Schagdarsurengin U Rastetter M Papritz M Hattenhorst UE. CpG island methylation and expression of tumour-associated genes in lung carcinoma. Eur J Cancer. (2005) 41:1223–36. 10.1016/j.ejca.2005.02.020 15911247

[17] Marsit CJ Houseman EA Christensen BC Eddy K Bueno R Sugarbaker DJ. Examination of a CpG island methylator phenotype and implications of methylation profiles in solid tumors. Cancer Res. (2006) 66:10621–9. 10.1158/0008-5472.CAN-06-1687 17079487

[18] Hulbert A Jusue-Torres I Stark A Chen C Rodgers K Lee B. Early detection of lung cancer using DNA promoter hypermethylation in plasma and sputum. Clin Cancer Res. (2017) 23:1998–2005. 10.1158/1078-0432.CCR-16-1371 27729459 PMC6366618

[19] Ritchie ME Phipson B Wu D Hu Y Law CW Shi W. limma powers differential expression analyses for RNA-sequencing and microarray studies. Nucleic Acids Res. (2015) 43:e47. 10.1093/nar/gkv007 25605792 PMC4402510

[20] Huang da W Sherman BT Lempicki RA. Bioinformatics enrichment tools: paths toward the comprehensive functional analysis of large gene lists. Nucleic Acids Res. (2009) 37:1–13. 10.1093/nar/gkn923 19033363 PMC2615629

[21] Ooki A Maleki Z Tsay JJ Goparaju C Brait M Turaga N. A panel of novel detection and prognostic methylated DNA markers in primary non-small cell lung cancer and serum DNA. Clin Cancer Res. (2017) 23:7141–52. 10.1158/1078-0432.CCR-17-1222 28855354

[22] Sandoval J Mendez-Gonzalez J Nadal E Chen G Carmona FJ Sayols S. A prognostic DNA methylation signature for stage I non-small-cell lung cancer. J Clin Oncol. (2013) 31:4140–7. 10.1200/JCO.2012.48.5516 24081945

[23] Yan Z Li J Xiong Y Xu W Zheng G. Identification of candidate colon cancer biomarkers by applying a random forest approach on microarray data. Oncol Rep. (2012) 28:1036–42. 10.3892/or.2012.1891 22752057

[24] Nelson HH Marsit CJ Christensen BC Houseman EA Kontic M Wiemels JL. Key epigenetic changes associated with lung cancer development: results from dense methylation array profiling. Epigenetics. (2012) 7:559–66. 10.4161/epi.20219 22522909 PMC3398985

[25] Hwang SH Kim KU Kim JE Kim HH Lee MK Lee CH. Detection of HOXA9 gene methylation in tumor tissues and induced sputum samples from primary lung cancer patients. Clin Chem Lab Med. (2011) 49:699–704. 10.1515/CCLM.2011.108 21480815

[26] Youwei Z Yuan Y Yang L Peiying Z Pingsheng C Sanyuan S An inverse interaction between HOXA11 and HOXA11-AS is associated with cisplatin resistance in lung adenocarcinoma. Epigenetics. (2019) 14:12 10.1080/15592294.2019.1625673 PMC6691981 31144606

[27] Keum JS Kong G Yang SC Shin DH Park SS Lee JH. Cyclin D1 overexpression is an indicator of poor prognosis in resectable non-small cell lung cancer. Br J Cancer. (1999) 81:127–32. 10.1038/sj.bjc.6690661 10487623 PMC2374356

[28] Meissner A Gnirke A Bell GW Ramsahoye B Lander ES Jaenisch R. Reduced representation bisulfite sequencing for comparative high-resolution DNA methylation analysis. Nucleic Acids Res. (2005) 33:5868–77. 10.1093/nar/gki901 16224102 PMC1258174

[29] Suzuki M Jing Q Lia D Pascual M McLellan A Greally JM. Optimized design and data analysis of tag-based cytosine methylation assays. Genome Biol. (2010) 11:R36. 10.1186/gb-2010-11-4-r36 20359321 PMC2884539

