# Supplementary material for: A Diagnostic Panel of DNA Methylation Biomarkers for Lung Adenocarcinoma
Source: Front Oncol. 2019 Dec 3;9:1281. doi: 10.3389/fonc.2019.01281 (PMC6901798; doi:10.3389/fonc.2019.01281)
Supplement: Supplementary file 1 [file Table_1.DOCX]

**Supplementary Table 1.** The clinical and pathological characteristics of 25 patients with LUAD

| **Patient number** | **Gender** | **Age** | **Tumor types** | **Tumor location** | **Tumor staging** |
| --- | --- | --- | --- | --- | --- |
| 1 | male | 73 | LUAD | right upper lobe | IIIa |
| 2 | male | 64 | LUAD | right upper lobe | Ib |
| 3 | male | 62 | LUAD | right upper lobe | Ia |
| 4 | female | 75 | LUAD | left upper lobe | Ia |
| 5 | female | 48 | LUAD | left upper lobe | IIIa |
| 6 | male | 59 | LUAD | right upper lobe | IIIb |
| 7 | female | 48 | LUAD | right upper lobe | Ia |
| 8 | female | 73 | LUAD | right lower lobe | Ia |
| 9 | male | 48 | LUAD | right upper lobe | IIIa |
| 10 | male | 73 | LUAD | right upper lobe | Ib |
| 11 | female | 63 | LUAD | right lower lobe | IIIb |
| 12 | male | 68 | LUAD | left upper lobe | Ia |
| 13 | male | 64 | LUAD | left lower lobe | IIIa |
| 14 | female | 64 | LUAD | left lower lobe | IIIa |
| 15 | male | 65 | LUAD | left upper lobe | IIIb |
| 16 | female | 66 | LUAD | left middle lobe | Ia |
| 17 | female | 58 | LUAD | right middle lobe | IIIa |
| 18 | male | 55 | LUAD | right upper lobe | IIIa |
| 19 | male | 57 | LUAD | right lower lobe | Ia |
| 20 | male | 76 | LUAD | left lower lobe | IIIa |
| 21 | male | 56 | LUAD | right upper lobe | IIIa |
| 22 | female | 56 | LUAD | right upper lobe | IIIa |
| 23 | male | 53 | LUAD | left upper lobe | IIIa |
| 24 | male | 64 | LUAD | right upper lobe | Ib |
| 25 | female | 73 | LUAD | left lower lobe | IIb |
